# A sensorimotor basis for facial expressivity differences in autism

**DOI:** 10.1162/IMAG.a.981

**Published:** 2025-11-10

**Authors:** Kimberly S. Bress, Jennifer Quinde-Zilbut, Alisa R. Zoltowski, Caitlin A. Convery, Brianna Lewis, Baxter P. Rogers, Simon Vandekar, Brittany Travers, Carissa J. Cascio

**Affiliations:** Vanderbilt Brain Institute, Vanderbilt University, Nashville, TN, United States; Department of Psychiatry and Behavioral Sciences, Vanderbilt University Medical Center, Nashville, TN, United States; University of Kansas, Life Span Institute, Lawrence, KS, United States; Vanderbilt University, Institute of Imaging Science, Vanderbilt University Medical Center, Nashville, TN, United States; Department of Biostatistics, Vanderbilt University Medical Center, Nashville, TN, United States; Department of Kinesiology and Waisman Center, University of Wisconsin-Madison, Madison, WI, United States

**Keywords:** facial expression, sensorimotor behavior, autism, functional connectivity, primary motor cortex (M1), primary somatosensory cortex (S1)

## Abstract

In autism, differences in the appearance, timing, and intensity of facial expressions are a major barrier to social communication. While disrupted sensorimotor feedback has been proposed as a potential contributing factor, the neural pathways linking sensory input to facial motor control are poorly understood even in the general population. In this study, we provide novel characterization of resting-state functional connectivity (rs-FC) between the facial regions of the primary somatosensory (S1) and motor (M1) cortices in both nonautistic and autistic individuals. We identify that rs-FC is somatotopically patterned for the lower but not upper face in both groups, mirroring known anatomical differences in corticomotor inputs to the upper versus lower face musculature. We independently replicate this patterning in a large, open-source neuroimaging dataset. Critically, we demonstrate that upper face actions are selectively diminished in autism, and that the relationship between sensorimotor connectivity and facial behavior diverges between autistic and nonautistic individuals. These findings offer the first direct evidence of a sensorimotor basis for altered facial expressivity in autism, challenging long-held assumptions about the underlying mechanisms of this communication barrier and pointing toward new targets for therapeutic intervention.

## 1 Introduction

Facial expressions are a critical mechanism of nonverbal communication and social affiliation (Barrett et al., 2019). Atypical facial expressivity is a common characteristic of autism and contributes to the social communication challenges that are diagnostic for autism (Guha et al., 2018; Keating & Cook, 2021). Specifically, autistic individuals produce facial expressions that are blunted or exaggerated in magnitude, shorter in duration, and less likely to be congruent with the expressions of social partners (Trevisan et al., 2018). Additionally, autistic individuals report higher rates of masking (Han et al., 2022; Miller et al., 2021), including the need to effortfully modify facial expressions during social interactions. Such disparities are present even when the reported internal emotional experience is similar to that of nonautistic peers (Butera et al., 2023; Gillespie-Lynch et al., 2017; J. M. Quinde-Zlibut et al., 2021), suggesting a difference that is specific to the outward expression of emotion.

Compared with facial expression *perception*, little work has been published addressing the etiology of atypical facial expression *production* in autism (Jaswal & Akhtar, 2019; Kapp et al., 2019; Kimber et al., 2024). This bias has created a significant knowledge gap regarding how autistic individuals’ emotions are understood by others and reinforces unsubstantiated assumptions about the relationship between emotional expression and the experience of emotions in autism. Moreover, it has led to an absence of evidence-based, autism-centered interventions targeting this critical communication barrier.

The production of facial expressions is a sophisticated sensorimotor behavior that involves precise contraction of the facial muscles guided by dynamic sensory feedback. Given that (i) facial expressions are a sensorimotor behavior, (ii) sensorimotor deficits are a core characteristic of autism (American Psychiatric Association, 2013), and (iii) other facial sensorimotor functions such as feeding (Elsayed et al., 2022; St John & Ausderau, 2024) and speech (Ma et al., 2024; Patel et al., 2019) are disrupted in autism, sensorimotor differences may contribute significantly to atypical facial expression behavior and represent a potential therapeutic target (Hannant, Cassidy, et al., 2016; Hannant, Tavassoli, et al., 2016; Mosconi & Sweeney, 2015). However, despite growing recognition that atypical sensorimotor processing may contribute significantly to the social and cognitive features of autism (Kapp, 2025), there is little published work directly examining the sensorimotor basis of atypical facial expression in autism. The goal of this work is to address this gap, beginning with the primary sensory and motor cortices.

The somatotopic organization of the primary somatosensory (S1) and somatomotor (M1) cortices is foundational to the understanding of how complex sensorimotor behaviors are executed and regulated. It is well established that M1 receives sensory information through direct, somatotopically patterned connectivity with S1. This reciprocal pathway allows sensory input to shape motor output (Petrof et al., 2015; Rocco-Donovan et al., 2011), and, in turn, allows motor activity to modulate sensory processing (Gale et al., 2021; Umeda et al., 2019; Zagha et al., 2013). Theoretical models of sensorimotor control, such as internal forward models (Wolpert & Flanagan, 2001; Wolpert et al., 1995) and corollary discharge (Blakemore et al., 2002; Crapse & Sommer, 2008), describe how this continuous feedback loop is essential to enabling precise, adaptive movements.

In autism, it is proposed that disruptions in connectivity between sensory and motor systems lead to atypical sensorimotor development, which contributes not only to isolated motor skill impairments but also to a cascade of social and cognitive impacts (Russo et al., 2025). Applied to facial expression, this framework suggests that disrupted coordination of sensory feedback and motor control may contribute to the atypical appearance, timing, and intensity of expressions often seen in autism—features that have traditionally, and perhaps inaccurately, been attributed to differences in how autistic individuals experience or understand emotions.

While disruption of S1-M1 connectivity is known to result in reduced motor adaption and impaired execution of coordinated sensorimotor behaviors (Edwards et al., 2019; Vidoni et al., 2010), the functional organization of this connectivity for the face is poorly mapped. This gap likely reflects an empirical focus on the cingulomotor pathways that control the emotional aspects of spontaneous facial expression, neglecting that nearly all socially driven expressions involve voluntary modulation of these spontaneous components (Berenbaum & Rotter, 1992; Fridlund, 1991; Gothard, 2014; Morecraft & Van Hoesen, 1998). Thus, even in nonautistic individuals, it is largely unknown how functional connectivity (FC) between S1 and M1 contributes to facial sensorimotor behaviors, especially the voluntary aspects of facial expression.

In this study, we utilized resting-state functional magnetic resonance imaging (rs-fMRI) to test the hypotheses that FC among the face areas of S1 and M1 is somatotopically organized, and that this organization differs in autism. To assess whether the observed results could be replicated in larger samples of nonautistic and autistic individuals, we then retested these hypotheses in rs-fMRI data from an open-source repository, the Autism Brain Imaging Data Exchange (ABIDE) (Di Martino et al., 2014). Finally, using a subset of participants with both rs-fMRI and behavioral data, we demonstrate a relationship between specific topologic features of cortical sensorimotor FC and facial expression behavior in an autistic sample. This work points toward a sensorimotor basis for facial expressivity differences in autism, challenging assumptions about the origins of these differences and opening doors to novel therapeutic targets.

## 2 Methods

### 2.1 Participant recruitment and clinical assessment

We collected rs-fMRI scans and a battery of behavioral measures from both autistic (AUT) and nonautistic (NA) individuals between the ages of 6 and 60 years old at Vanderbilt University Medical Center (VUMC). Individuals were recruited across multiple parent fMRI cohorts; prior reports on these data have been published by Cascio et al. (2014) and Failla et al. (2020). All procedures were approved by the Institutional Review Board for Human Subjects at VUMC and conducted in accordance with the guidelines and regulations on ethical human research set forth in the Declaration of Helsinki. All participants provided written, informed consent for all study activities, including tasks involving video recording. Participants under the age of 18 years provided informed assent, and their legal guardian provided informed consent. All participants were compensated $20 per hour for their time.

Autism diagnoses were confirmed by a licensed psychologist specializing in the assessment of Autism Spectrum Disorder, including use of research-reliable administration of the Autism Diagnostic Observation Schedule-2 (ADOS-2) (Lord et al., 1999), algorithm items from the Autism Diagnostic Interview-Revised (ADI-R) (Le Couteur et al., 1989), and global clinical impression. Exclusion criteria for both NA and AUT participants in this study were (1) age below 6 years or above 60 years, (2) Full Scale IQ (FSIQ) < 60, as determined using the Wechsler Abbreviated Scale of Intelligence Second Edition (WASI-II) (Wechsler, 1999), (3) diagnosis of other neurologic or genetic disorders, (4) psychiatric diagnosis within the past 5 years other than ADHD, anxiety, or depression, (5) sensory impairments unrelated to autism diagnosis (e.g., uncorrected visual or hearing impairment), (6) substance use disorder or dependence during the 2 years before study enrollment, and (7) current facial paralysis due to neurologic, psychogenic, or exogenous (e.g., botulinum toxin injection) causes. Additional exclusion criteria for the NA participants in this study included history of cognitive or sensory impairment, use of psychotropic medications other than selective serotonin reuptake inhibitors (SSRIs), or clinically elevated scores on the Social Communication Questionnaire (SCQ).

### 2.2 Sample characteristics

**Fig. 1.**
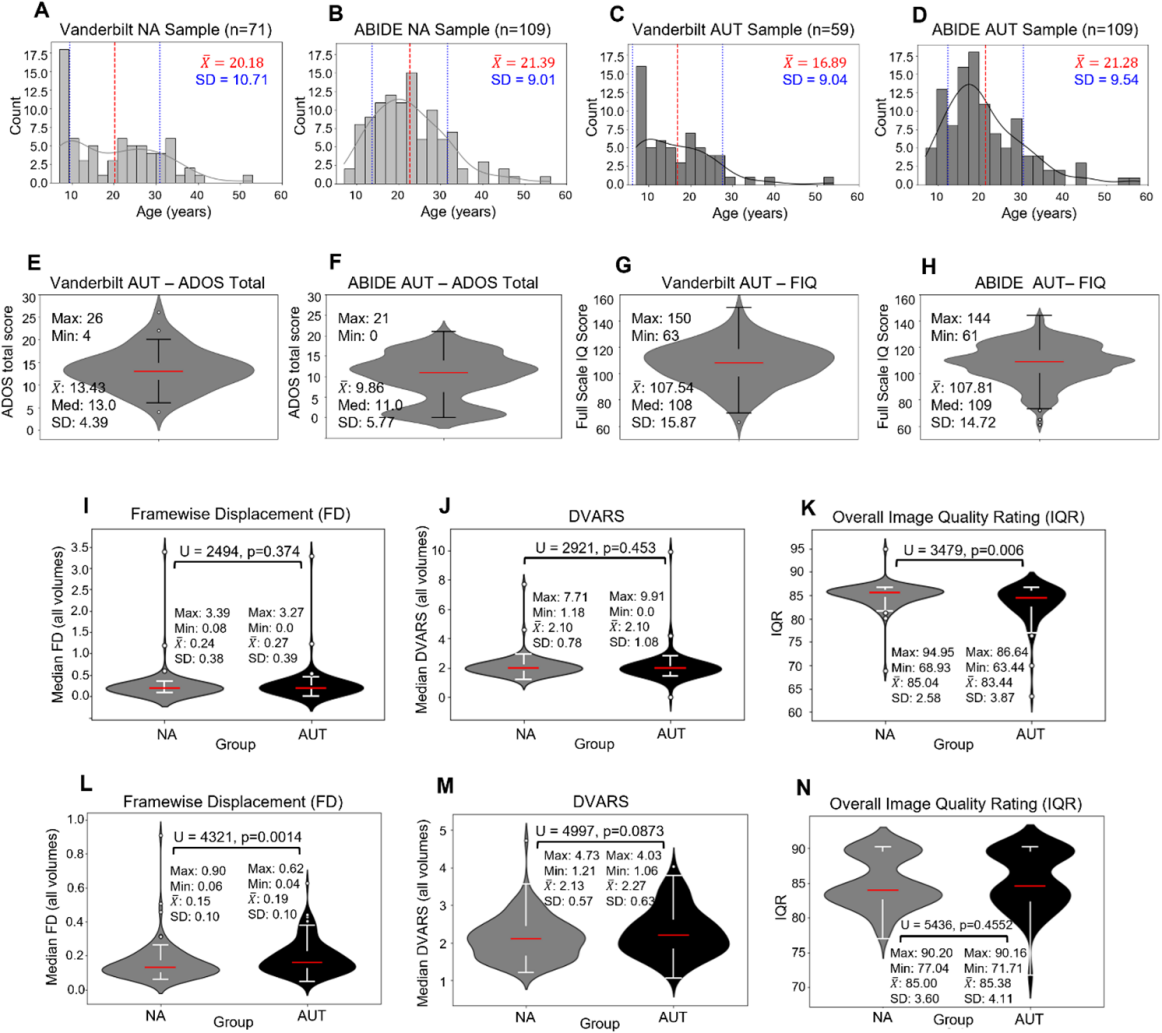
Age distributions, clinical characterization, and QA metrics—Vanderbilt and ABIDE samples. (A–D) The distribution of participant ages across groups from the Vanderbilt (1A-NA, 1C-AUT) and ABIDE (1B-NA, 1D-AUT) samples. Age distributions did not significantly differ across groups within samples (Vanderbilt: U = 2475, p = 0.0751; ABIDE: U = 6489, p = 0.1187). Across samples, age distribution also did not differ for the NA group (U = 3173.0, p = 0.0776). However, for the AUT group, the Vanderbilt sample was significantly younger than the ABIDE sample (U = 2265.0, p = 0.0015). (E and F) The distribution of ADOS total scores in the Vanderbilt (E) and ABIDE (F) AUT samples. (1 and H) The distribution of full-scale IQ scores (FIQ) in the Vanderbilt (G) and ABIDE (H) samples. (I–K) The distribution of QA metrics across groups in the Vanderbilt sample. While the NA and AUT groups did not differ in framewise displacement (FD) (U = 2494, p = 0.374) or the temporal derivative variance across voxels (DVARS) (U = 2921, p = 0.453), which are measures of motion, the NA group had significantly higher Image Quality Ratings (IQR) (U = 3479, p = 0.006). (L–N) The distribution of QA metrics across groups in the ABIDE sample. While the NA and AUT groups did not differ in DVARS (U = 4997, p = 0.0873) or IQR (U = 5436, p = 0.4552) metrics, the AUT group did have significantly higher FD (U = 4321, p = 0.0014).

We collected rs-fMRI data from a total of n = 208 individuals, including n = 92 AUT and n = 116 NA individuals. We then performed quality assessment (QA) of rs-fMRI data (see “fMRI preprocessing and quality assessment”) to achieve a final sample size of n = 130, including n = 59 (42 male, 17 female—$\bar{x}$ = 16.89 years) AUT and n = 71 (51 male, 20 female—$\bar{x}$ = 20.18 years) NA individuals. There was no significant difference between diagnostic groups in age distributions (U = 2475.0, p = 0.075; Fig. 1A, C), with an overall age range of 7–54 years in the full sample. There was also no significant difference between groups in the proportion of individuals excluded after QA.

### 2.3 Neuroimaging data acquisition

All rs-fMRI data were acquired on a 3 Tesla Philips Achieva MRI scanner with a 32-channel SENSE head coil. Whole-brain EPI T2*-weighted functional images were acquired using axial slices with an isotropic 2.5 mm^3^ voxel size (TR = 2 s, TE = 25 msec, flip angle = 90°, acquisition matrix = 96 x 96, no gap). A high-resolution T1-weighted scan (TR = 8 msec, TE = 3.7 msec, acquisition matrix: 256 x 256, 1 mm^3^ resolution; 6’30” scan time) was collected to provide a template for functional image registration. During the scan, participants were instructed to keep their eyes open and focus on a fixation cross.

### 2.4 fMRI preprocessing and quality assessment

We preprocessed all rs-fMRI data using the connprep pipeline developed by coauthor BPR (https://github.com/baxpr/connprep), including head motion realignment (SPM12 two-stage) and production of mean fMRI, rigid body coregistration of mean fMRI to the skull-stripped, bias-corrected T1 structural image produced by the Computational Anatomy Toolbox in SPM12 (CAT12) (Gaser et al., 2023), computation of volume quality metrics including framewise displacement (FD), and the temporal derivative variance across voxels (DVARS), slicing of realigned fMRI to native space and warping to MNI space via CAT12 transform, removal of confounds from native and MNI space fMRIs via simultaneous regression (0.01–0.10 Hz bandpass filter, 6 estimated motion parameters and their first differences, and 6 principal components from the white matter and CSF compartments), and removal of mean signal from the gray matter compartment. Although global signal regression (GSR) raises concerns about distance-dependent artifacts (Ciric et al., 2017; Gotts et al., 2013), large-scale evaluations have shown that GSR improves data quality across various motion exclusion thresholds. Specifically, when utilized with GSR, aCompCor or ICA-AROMA more effectively minimize the number of significant connections driven solely by motion levels and thus reduce motion-related bias in FC (Parkes et al., 2018).

After preprocessing, we performed QA of rs-fMRI for all participants. Participants were excluded based on a priori thresholds for computed measures of motion artifact and distortion including median FD >0.5 mm (n = 7), volumes with DVARS >5% or FD >1 mm accounting for over 20% of total scan time (n = 7), and/or an overall Image Quality Rating (IQR) less than 70 (n = 5). The IQR is computed via the CAT12 toolbox from both signal-to-noise ratio and bias measurement; it is scaled from 0 (low quality) to 100 (high quality) and interpreted on a letter grade scale as reported by Gaser et al. (2023). The distributions of FD, DVARS, and IQR across both groups are shown in Figure 1I–K. Additionally, 50 participants were excluded for unresolvable preprocessing errors (most often due to missing or incomplete data) and nine participants were excluded due to poor coregistration of structural and functional images on visual inspection by trained personnel (coauthors A.R.Z., K.S.B.).

### 2.5 Pairwise Region of Interest (ROI)-based functional connectivity analysis

**Fig. 2.**
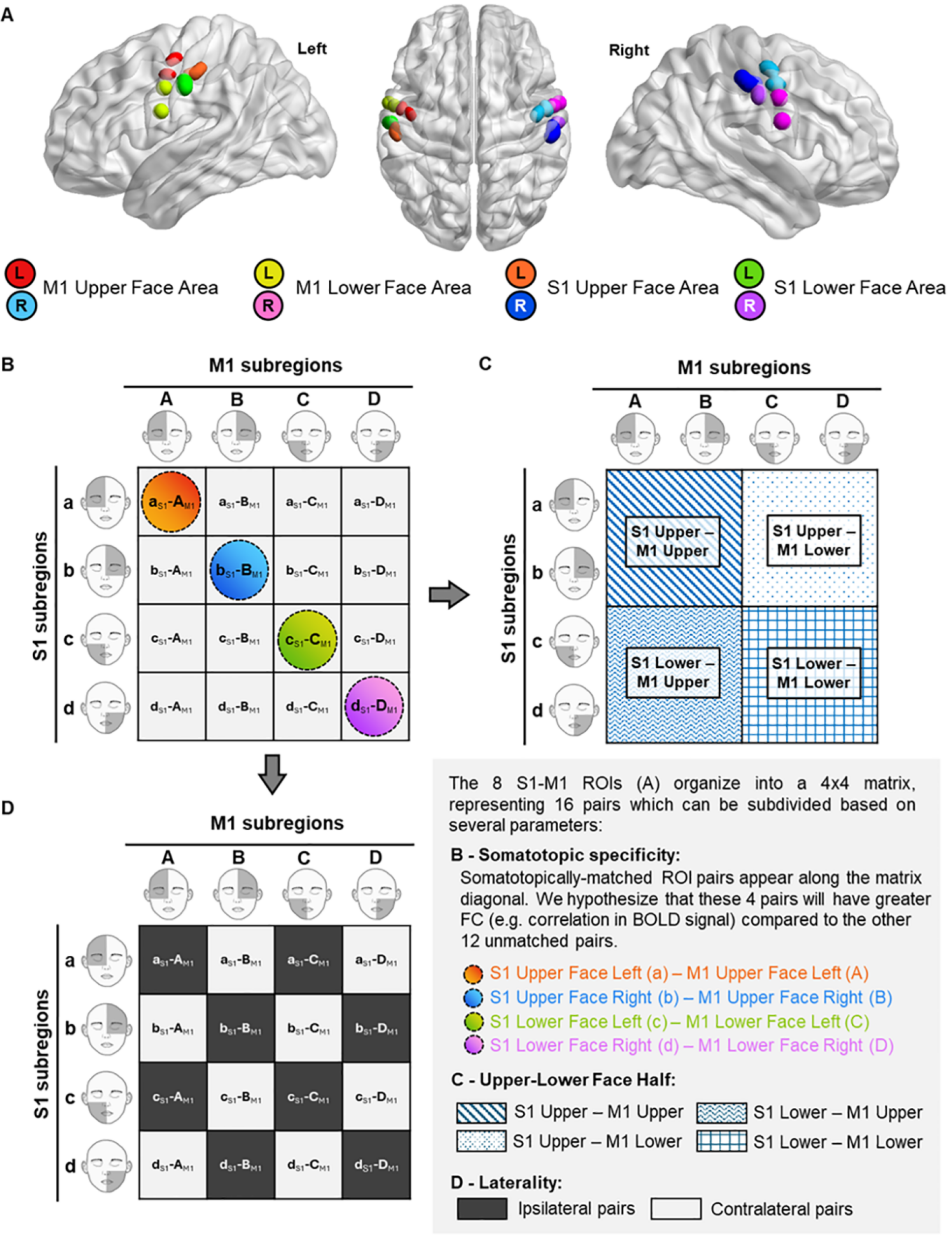
Representation of the primary somatosensory (S1) and somatomotor (M1) regions of interest. The regions of interest (ROI) representing the somatotopic upper and lower face areas of the S1 and M1 cortices are shown in (A). (B) The organization of these 8 ROIs into a 4 x 4 matrix representing 16 S1-M1 ROI pairs, as constructed for the pairwise FC analysis. The subdivision of these ROI pairs based on the parameters of somatotopic specificity (B), upper–lower face half (C), and hemispheric laterality (D) are illustrated, with color and texture coding shown in the legend. Comparison across these subdivisions is necessary to evaluate if FC is somatotopically organized (B), recapitulates neuroanatomic differences in upper versus lower face motor control (C), and is lateralized (D), as most expressions are symmetric across the right and left face.

We performed a seed-based resting-state connectivity analysis to interrogate pairwise connections between the upper and lower somatotopic face areas of S1 and M1. S1 and M1 somatotopic regions of interest (ROI) were generated from mean MNI coordinates for body-part-specific electrostimulation responses mapped by F.-E. Roux et al. (2018) (S1) and (2020) (M1). We generated 4 mm spheres around the mean coordinates reported for each distinct body part (Supplementary Table S1); these spheres were then organized into body region groups and added to create the composite ROIs shown in Figure 2A, which can be accessed at https://github.com/bressks1/s1_m1_face_roi_masks. The spherical S1 and M1 ROIs were confirmed to fall completely within the precentral gyrus and postcentral gyrus, respectively, using the Harvard-Oxford Cortical Atlas implemented in FSL (Jenkinson et al., 2012). Individual participant FC maps and matrices were computed using the conncalc pipeline developed by BPR (https://github.com/baxpr/conncalc). The mean time series was extracted from the fMR image for each of the eight ROIs. ROI-to-ROI connectivity matrices including both the Pearson’s R correlation coefficients and Fisher-Z transformations were then computed, as well as a voxel-wise connectivity Z map.

### 2.6 Replication in the Autism Brain Imaging Exchange Dataset (ABIDE)

This study includes a replication of analyses in the Autism Brain Imaging Data Exchange I (ABIDE I). The ABIDE, described in detail by Di Martino et al. (2014), is a publicly available, post hoc repository of rs-fMRI, structural MRI, and basic phenotypic data from both AUT and NA individuals across 17 international sites. Available phenotypic data include demographic information (e.g., age, sex), as well as total scores for clinical assessments including the Autism Diagnostic Observation Schedule (ADOS) (Lord et al., 1999), Autism Diagnostic Interview-Revised (ADI-R) (Le Couteur et al., 1989), and an age-appropriate full-scale IQ measure.

We utilized data from 5 of the 17 ABIDE sites (CMU, Caltech, USM, Pitt, and Yale), selected based on their common acquisition parameters including Siemens scanner manufacturer, Repetition Time (TR)< = 2000 ms, interleaved slice acquisition order, and anterior-to-posterior (A>>P) phase encoding direction. Rs-fMRI, phenotypic, and demographic data for these n = 278 participants (27 CMU, 38 Caltech, 101 USM, 57 Pitt, and 55 Yale) were retrieved from the NeuroImaging Tools and Resource Collaboratory (NITRC, https://www.nitrc.org/). Rs-fMRI data were preprocessed using the connprep pipeline and QA was performed as described in “fMRI preprocessing and quality assessment,” including exclusion of participants based on the previously stated thresholds for FD (n = 16), DVARS (n = 2), and IQR (n = 0). The distributions of median FD, DVARS, and IQR across both groups from the ABIDE are shown in Figure 1L–N. In addition, raw T1 images were manually inspected for gross distortions and raw fMR images were manually inspected for field-of-view errors (n = 20), signal dropout (n = 17), and other gross errors or distortions (n = 5). This led to a final sample of n = 109 (102 male, 7 female) AUT and n = 109 (97 male, 12 female) NA individuals, with no significant difference in overall age distributions across groups (U = 6489, p = 0.1187; Fig. 1B, D). The conncalc pipeline was then used as described in “fMRI preprocessing and quality assessment” to compute FC maps and matrices for each participant, using the same ROIs.

### 2.7 Behavioral measure of facial expressivity

**Fig. 3.**
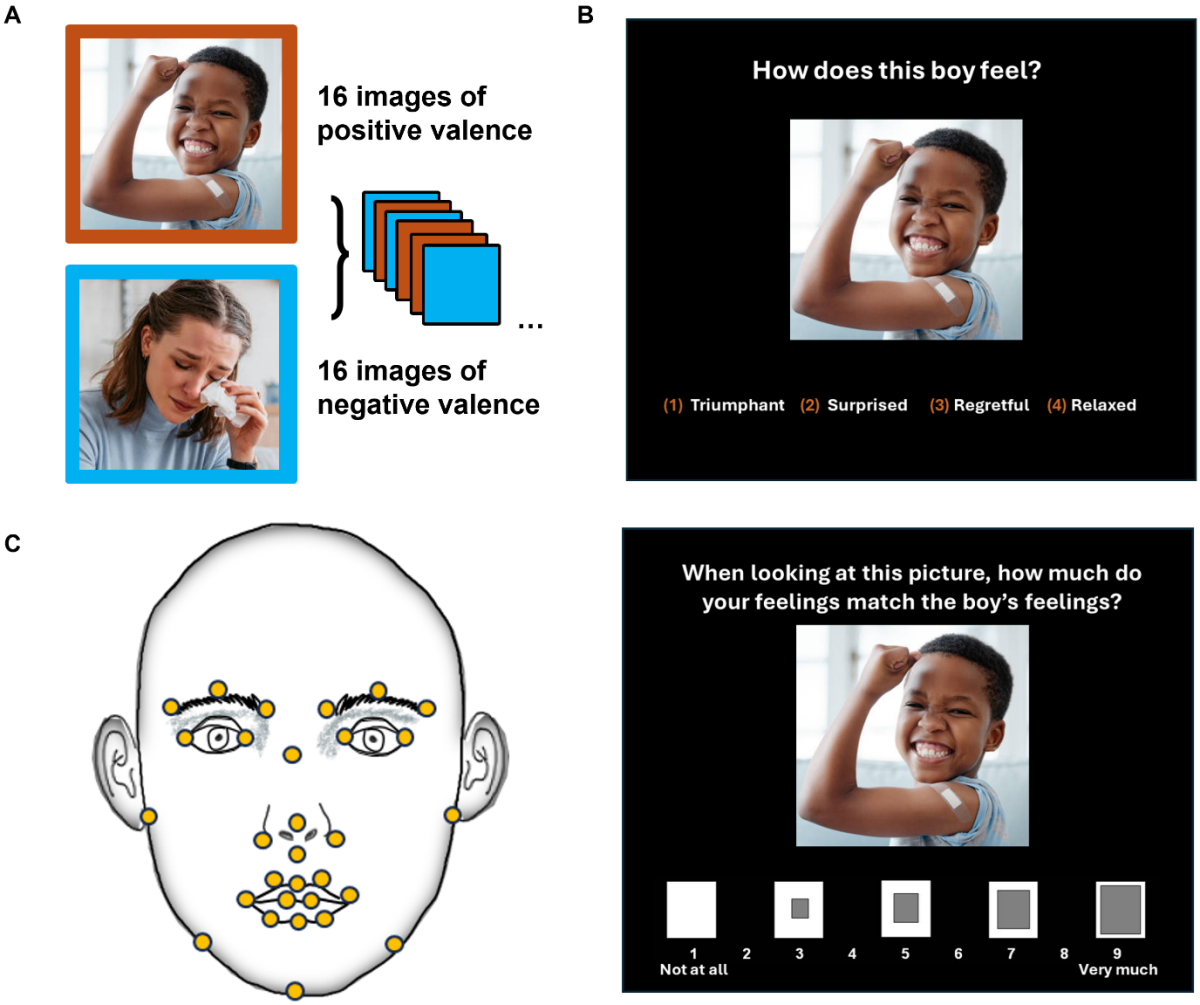
Behavioral measure of facial expressivity—Automated facial action coding during the MET-J. In the MET-J task, participants passively view a series of 32 emotionally charged images selected from the International Affective Picture System (IAPS), including 16 images of positive valence and 16 images of negative valence (A). For each image, participants are asked to identify the depicted emotion from a multiple choice array and rate the extent to which they share or feel the depicted emotion on a Likert scale from 1 (“Not at all”) to 9 (“Very much”) (B). During completion of the task, movement of the major facial landmarks (C) is detected and quantified by the iMotions v.6 software AffDex engine as discrete facial action units based on the Facial Action Coding System (FACS) (Ekman & Friesen, 1978).

Of the 130 individuals in the Vanderbilt sample, 36 (17 AUT, 19 NA; 8–34 years, $\bar{x}$ = 19.86 years) completed a behavioral measure of facial expressivity: the Multifaceted Empathy Test–Juvenile (MET-J) (Poustka et al., 2010). The original MET (Dziobek et al., 2008; J. M. Quinde-Zlibut et al., 2021) is a 50-trial computer-based task validated for measuring cognitive and emotional empathy; the MET-J is an abbreviated, 32-trial version validated for use in school-age children as young as 8 years old. In each trial, participants view a still photograph depicting a person with an emotional facial expression, surrounded by a contextual background. Across all trials, there are an equal number of photographs with positive (25 MET; 16 MET-J) and negative (25 MET; 16 MET-J) emotional valence, presented in a random order. All images are drawn from the International Affective Picture System (IAPS), a validated database of photographs that is widely used for standardized testing of emotion (Fig. 3A) (Lang et al., 1997).

After viewing each photograph ad libitum, participants immediately complete a sequence of self-paced response questions. This includes (1) labeling the emotion displayed in the image by selecting one option from four forced-choice labels; after selection, the participant is shown the correct emotion label, and (2) rating how much they share the emotion of the depicted person on a Likert scale from 1 (“Not at all”) to 9 (“Very much”) (Fig. 3B). These response items are conceptualized as measures of cognitive empathy (emotion recognition accuracy) and emotional empathy (emotion sharing or emotional resonance), respectively. Notably, we modified this original design by reversing the order of the cognitive empathy and emotional empathy response items. This modification was made to ensure that emotional empathy responses reflected participants’ immediate reactions to the image, rather than being influenced by feedback from the cognitive empathy response (J. Quinde-Zlibut et al., 2022).

To complete the MET-J, participants were seated at a laptop computer workstation in a quiet, well-lit testing room. Using the iMotions v.6 software platform (*iMotions Biometric Research Platform, version 6.1*, *iMotions, 2021*) paired with a webcam (Logitech c920x Pro HD, video frame rate: 30 Hz), we recorded participants’ facial movements while they completed the MET-J. Participants were explicitly informed that they would be video recorded during study tasks and provided their informed consent or assent prior to participation. At completion of the task, facial video recordings were exported for post hoc processing using the iMotions v.6 AffDex SDK (McDuff et al., 2016). The AffDex engine classifies 18 types of facial movements, called “facial action units”, based on the Facial Action Coding System (FACS) developed by Ekman and Friesen (1978). The distinct facial action units (AUs, e.g., “upper lip raise”, “outer brow raise”) reflect contractions of the muscles of facial expression and are identified by tracking and analyzing the movement of anatomic landmarks across the face (Fig. 3C). At a sampling rate of 60 Hz, the AffDex algorithm computes likelihood scores representing the probability (0–100%) that a detected AU would be coded by a manual human rater. For each participant, we additionally computed emotion recognition accuracy as the percentage of images for which the participant correctly identified the depicted emotion from the multiple-choice array and emotional resonance as the average of shared emotion ratings across the 32 stimuli.

## 3 Hypotheses and Statistical Approach

Using rs-fMRI data from the Vanderbilt (n = 130, 59 AUT) and ABIDE (n = 218, 109 AUT) samples, we used linear mixed-effects models to test the following series of hypotheses. All models were estimated in R using the *lmer* function from the lme4 package (Bates et al., 2015), fitted by restricted maximum likelihood (REML) (Corbeil & Searle, 1976). Significance testing for fixed effects was conducted using t-tests with Satterthwaite’s approximation for degrees of freedom. When significant interactions between fixed effects were identified, we used the *emmeans* package (Lenth, 2017) to compute estimated marginal means (EMMs). These EMMs provide model-based adjusted means that account for the influence of other covariates and were utilized to probe and interpret the specific nature of the observed interaction effects.

Below, we describe the hypothesis-specific fixed effects included in each model. Where appropriate, a random intercept was fitted for each participant; for the ABIDE sample, we included a random intercept for participant nested within site. Additionally, all ABIDE models included a random effect for “eye status” (i.e., whether participants’ eyes were open [3 sites; CMU, USM, Yale] or closed [3 sites; Caltech, Pitt, Yale]).

Motion parameters were not included as fixed effects, as motion-related QA metrics (e.g., FD, DVARS) were matched across groups, and the six estimated motion parameters and their first differences were included as regressors in preprocessing for all participants (“fMRI preprocessing and quality assessment”).

The total and residual variance estimates for all models, as well as the variance explained by random effects, are reported in Supplementary Tables S2 (Vanderbilt) and S3 (ABIDE).

### 3.1 Linear modeling of mixed effects on S1-M1 functional connectivity: Somatotopic organization

Since other body areas involved in complex sensorimotor behaviors exhibit somatotopically organized S1-M1 connectivity, we hypothesized that (1) in the NA sample, FC (e.g., BOLD signal correlation) would be stronger for somatotopic S1-M1 ROI pairs than for non-somatotopic pairs (Fig. 2B) and (2) AUT individuals would show reduced somatotopic specificity in S1-M1 FC compared with NA individuals.

To test these hypotheses, we first classified each of the 16 ROI pairs as either somatotopically matched (e.g., Left S1 Upper Face—Left M1 Upper Face, Right S1 Upper Face—Right M1 Upper Face, Left S1 Lower Face—Left M1 Lower Face, Right S1 Lower Face—Right M1 Lower Face) or non-somatotopically matched. We then fit a linear mixed effect model on the Fisher-transformed Pearson’s correlations (z-values) representing FC for each ROI pair. Fixed effects included diagnostic group (NA or AUT), somatotopic matching (matched vs. unmatched), age, and gender (Eq. (1), Fig. 2B). To account for repeated measures (16 ROI pairs per participant), we included participant as a random intercept, either alone (Vanderbilt) or nested within site (ABIDE).

$$\begin{array}{l} \text{FC }\sim \text{ group}\;+\;\text{somatotopic matching}\;\\ \;\;\;+\;\text{age}\;+\;\text{gender}\;+\;\left(1|\text{ participant}\right) \end{array}$$ Eq. (1)

### 3.2 Linear modeling of mixed effects on S1-M1 functional connectivity: Upper versus lower face

As it is well documented in both humans and nonhuman primates that primary motor control of the upper and lower halves of the face involves distinct neuroanatomic pathways with different balances of contralateral versus ipsilateral input (Bress & Cascio, 2024), we hypothesized that (1) S1-M1 pairs representing the same somatotopic face half (e.g., S1 Upper Face—M1 Upper Face, S1 Lower Face—M1 Lower Face) would show stronger FC than pairs representing different halves (e.g., S1 Upper Face—M1 Lower Face, S1 Lower Face—M1 Upper Face), and (2) this specificity would be reduced in autism.

To test these hypotheses and evaluate the effects of hemispheric laterality, we first classified each ROI pair by its face half combination (Fig. 2C): (1) S1 Upper—M1 Upper, (2) S1 Upper—M1 Lower, (3) S1 Lower—M1 Lower, or (4) S1 Lower—M1 Upper. Each pair was also labeled as either ipsilateral (ROIs within the same hemisphere) or contralateral (ROIs within different hemispheres) (Fig. 2D). We then fit a linear mixed-effects model on FC (z-value), with fixed effects of diagnostic group (NA or AUT), face half combination, laterality, age, and gender. Again, we included participant as a random intercept, either alone (Vanderbilt) or nested within site (ABIDE).

$$\begin{array}{l} \text{FC }\sim \text{ group}\;+\;\text{face half combination}\;+\;\text{laterality}\\ \;\;\;\;\;+\text{ age}\;+\;\text{gender}\;+\;\left(1|\text{ participant}\right) \end{array}$$ Eq. (2)

### 3.3 Linear modeling of mixed effects on facial expression behavior

From the MET-J task, we obtained the following output metrics for each participant, for each of the 32 stimuli: (1) the participant’s rating of how much they shared the emotion depicted in the stimulus (Likert scale, 1 to 9); (2) whether the participant correctly identified the depicted emotion in the 4 option forced-choice question; and (3) likelihood scores for all 18 AUs, sampled at 60 Hz from stimulus onset through completion of the final response item.

To assess group differences in emotion recognition accuracy, we fit a linear mixed-effects model on each participant’s percentage of correctly identified emotions, including group, gender, and age as fixed effects (Eq. 3a). To assess group differences in emotional resonance, we fit a linear mixed-effects model on the shared emotion ratings across all 32 stimuli, including group, age, and gender as fixed effects, and accounting for repeated measures by fitting random intercepts for both participant and stimulus. Although the emotion ratings were on a Likert scale ranging from 1 to 9, we treated them as a continuous variable, which is a common and accepted approach for point scales with five or more response options.

$$\begin{array}{l} \text{Percent correctly identified emotions }\sim \mathrm{group}\;\\ \;\;\;+\;\text{age}\;+\;\text{gender} \end{array}$$ Eq. (3a)

$$\begin{array}{l} \text{Shared emotion rating }\sim \text{ group}\;+\;\text{age}\;+\;\text{gender }\\ \;\;\;+\left(1|\text{ stimulus}\right)\;+\;\left(1|\text{ participant}\right) \end{array}$$ Eq. (3b)

To assess group differences in facial expression behavior, we used the following approach. For each participant, stimulus, and AU, we computed the integral of the AU likelihood score over time, yielding the area under the curve (AUC). We then log-transformed these AUC values (log(AUC)) to address their substantial positive skew, heteroscedasticity, and wide range spanning multiple orders of magnitude. For each participant, this procedure yielded one log(AUC) value per AU for each of the 32 stimuli. We then categorized each of the 18 AUs as belonging to either the upper face (8, e.g., AU4: Brow Furrow) or lower face (10, e.g., AU20: Lip Stretch; see Fig. 8). We then fit a linear mixed-effects model on the log(AUC) values from all stimuli and AUs. Fixed effects included diagnostic group, the AU face half (upper vs. lower), and their interaction, as well as age, gender, emotion recognition accuracy, and average emotional resonance rating across all stimuli. To account for repeated measures (32 stimuli per participant, 18 AUs per stimulus), we modeled participant, stimulus, and AU as random intercepts. Finally, to examine the relationship between facial expression behavior and S1-M1 FC, we re-estimated this model, adding FC (z-values for all S1-M1 ROI pairs) as a fixed effect, along with a three-way interaction among diagnostic group, AU face half, and FC. To account for repeated measures introduced by the 16 unique ROI pairs per participant, we included an additional random intercept for ROI pair.

$$\begin{array}{l} \text{log}\left(\text{AUC}\right)\sim \text{ group}*\text{AU face half}\;+\text{ age}\;+\;\text{gender}\;\\ \;\;\;+\;\text{recognition accuracy}\;+\text{ resonance rating}\;\\ \;\;\;+\;\left(1|\text{ participant}\right)\;+\;\left(1|\text{ stimulus}\right)\;+\;\left(1|\text{ AU}\right) \end{array}$$ Eq. (4)

$$\begin{array}{l} \text{log}\left(\text{AUC}\right)\sim \text{ group}*\text{AU face half}*\text{FC}\;+\;\text{age}\;+\;\text{gender}\;\\ \;\;\;+\text{ recognition accuracy}\;+\;\text{resonance rating}\;\\ \;\;\;+\;\left(1|\text{ participant}\right)\;+\;\left(1|\text{ stimulus}\right)\;+\;\left(1|\text{ AU}\right)\;\\ \;\;\;+\;\left(1|\text{ ROI pair}\right) \end{array}$$ Eq. (5)

Notably, while prior analyses of iMotions AffDex data (J. Quinde-Zlibut et al., 2022) have used measures of central tendency (the mean or median AU likelihood score across the stimulus duration) to operationalize facial expressivity, our approach of integrating AU likelihood score over time captures the cumulative magnitude over time. This provides a more comprehensive measure of how facial actions dynamically occur across the stimulus period and is less sensitive to skew and periods of inactivity (zero-inflation) compared with a single average or median value.

## 4 Results

### 4.1 Cortical sensorimotor connectivity is somatotopically organized, but only for the lower face

**Fig. 4.**
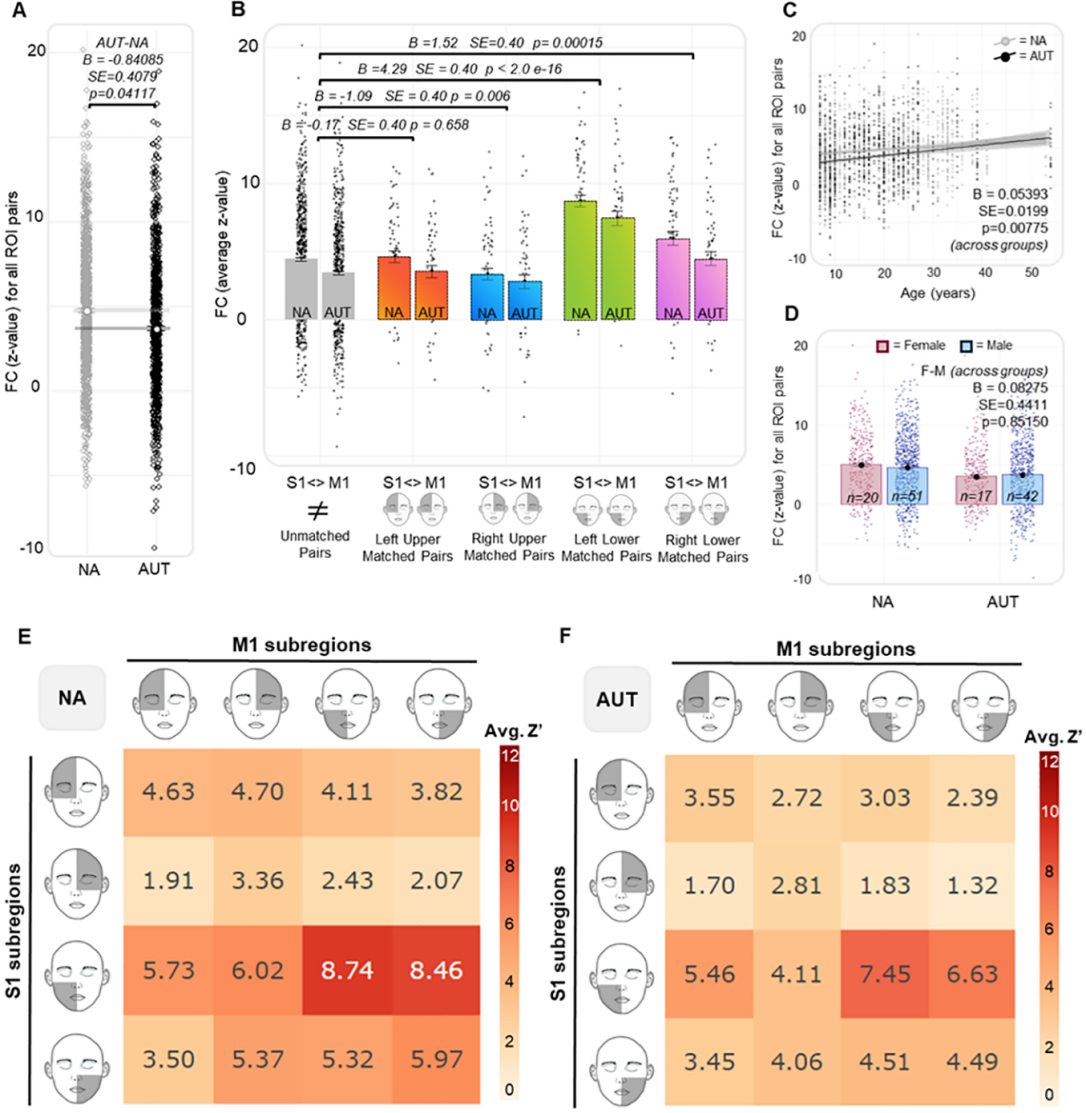
Vanderbilt Sample—Effect of group, somatotopy, and demographic characteristics on S1-M1 functional connectivity. (A) The main effect of group on functional connectivity (FC, e.g., z-value) for all 16 S1-M1 ROI pairs. (B) The difference in FC across the somatotopically matched ROI pairs and non-somatotopically matched ROI pairs. (C) The main effect of age on FC across groups; the reported contrast estimate (B = 0.0539) is for the main effect of age on FC. (D) The main effect of gender on FC across groups; the reported contrast estimate (B = 0.08275) is for the main effect of gender on FC. (E and F) Heatmaps (NA) and (AUT) illustrate the average z-value for each S1-M1 ROI pair, with ROI pairs along the diagonal representing somatotopically matched ROIs. In these heatmaps, redder colors represent stronger FC (larger average z value), while whiter colors represent weaker FC (smaller average z values).

As described, we used linear mixed models to investigate differences in FC across groups and between somatotopic and nonsomatotopic ROI pairs in the Vanderbilt sample. Autism diagnosis was associated with reduced FC (FC ~ group + somatotopic identity + age + gender + (1 | participant); [AUT-NA] B = -0.8408, SE = 0.4079, p = 0.0411; Fig. 4A). For the lower face, somatotopic ROI pairs demonstrated greater FC than non-somatotopic pairs in both the left (B = 4.29, p < 2.0e-16) and right (B = 1.52, p = 0.0001) hemispheres (Fig. 4B). Conversely, for the upper face, somatotopic ROI pairs demonstrated less FC than nonsomatotopic pairs in the right hemisphere (B = -1.09, p = 0.0063) but no significant difference from nonsomatotopic pairs in the left hemisphere (B = 0.177, p = 0.6581) (Fig. 4B). Increasing age was associated with greater FC (B = 0.0539, SE = 0.0199, p = 0.0077; Fig. 4C), while there was no significant difference in FC across genders ([F-M] B = 0.0827, SE = 0.4411, p = 0.8515; Fig. 4D). To assess whether FC across the somatotopic and non-somatotopic pairs differed across groups, we re-ran the prior model including an interaction term between diagnostic group and somatotopic identity (FC ~ group*somatotopic identity + age + sex + (1 | participant). There was no significant interaction (Supplementary Table S4). Additionally, inclusion of the interaction term resulted in a worsened model fit, as represented by higher Akaike Information Criterion (AIC) (11076.87 < 11080.18) and Bayesian Information Criterion (BIC) (11133.27 < 11159.14) values (Supplementary Table S3).

**Fig. 5.**
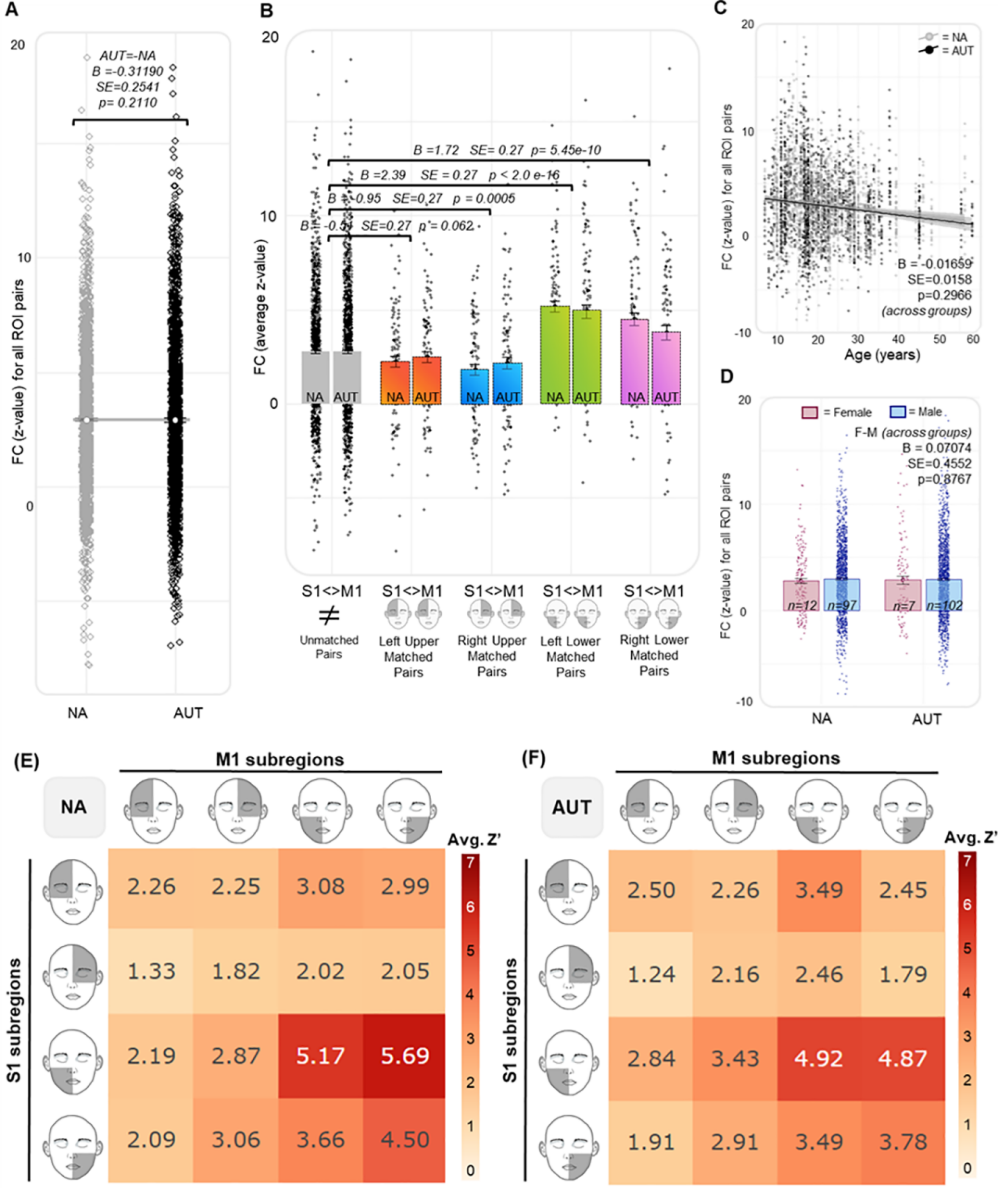
ABIDE Replication—Effect of group, somatotopy, and demographic characteristics on S1-M1 functional connectivity. Figure represents a replication of the results shown in Figure 4. (A) The main effect of group on functional connectivity (FC, e.g., z-value) for all 16 S1-M1 ROI pairs. (B) The difference in FC across the somatotopically matched ROI pairs and non-somatotopically matched ROI pairs. (C) The main effect of age on FC across groups; the reported contrast estimate (B = -0.01659) is for the main effect of age on FC. (D) The main effect of gender on FC across groups; the reported contrast estimate (B = 0.07074) is for the main effect of gender on FC. (E and F) Heatmaps (NA) and (AUT) illustrate the average z-value for each S1-M1 ROI pair, with redder colors representing stronger FC (larger average z-value). While the overall pattern of these heatmaps reflects that which was observed in the Vanderbilt sample, the relative magnitude of the average z-values is reduced, as reflected in the decreased maximum value of the color scale.

When this analysis was repeated in the ABIDE sample, we again observed that for the lower face, somatotopic ROI pairs had significantly greater FC than non-somatotopic ROI pairs in both the left (B = 2.39, p < 2.0e-16) and right (B = 1.72, p = 5.45e-10) hemispheres (Fig. 5B). Additionally, for the upper face, somatotopic ROI pairs had significantly less FC than non-somatotopic pairs in the right hemisphere (B = -0.95, p = 0.0005), but were not significantly different from non-somatotopic pairs in the left hemisphere (B = -0.51, p = 0.062). Unlike the Vanderbilt sample, there was no significant main effect of group on FC ([AUT-NA] B = -0.31190, SE = 0.2541, p = 0.2110; Fig. 5A). Additionally, there was no significant effect of age on FC (B = -0.01659, SE = 0.01584, p = 0.2966; Fig. 5C) and no significant contrast observed across genders ([F-M] B = 0.07074, SE = 0.4552, p = 0.8767; Fig. 5D). Consistent with the Vanderbilt sample, when we added an interaction term between somatotopic identity and diagnostic group, there was no significant interaction (Supplementary Table S4), and inclusion of the interaction term resulted in a worsened model fit, represented by higher AIC (17148.47 < 17151.5) and BIC (17222.19 < 17249.79) values.

### 4.2 S1-M1 connectivity differs across the upper and lower face and may be attenuated in autism

**Fig. 6.**
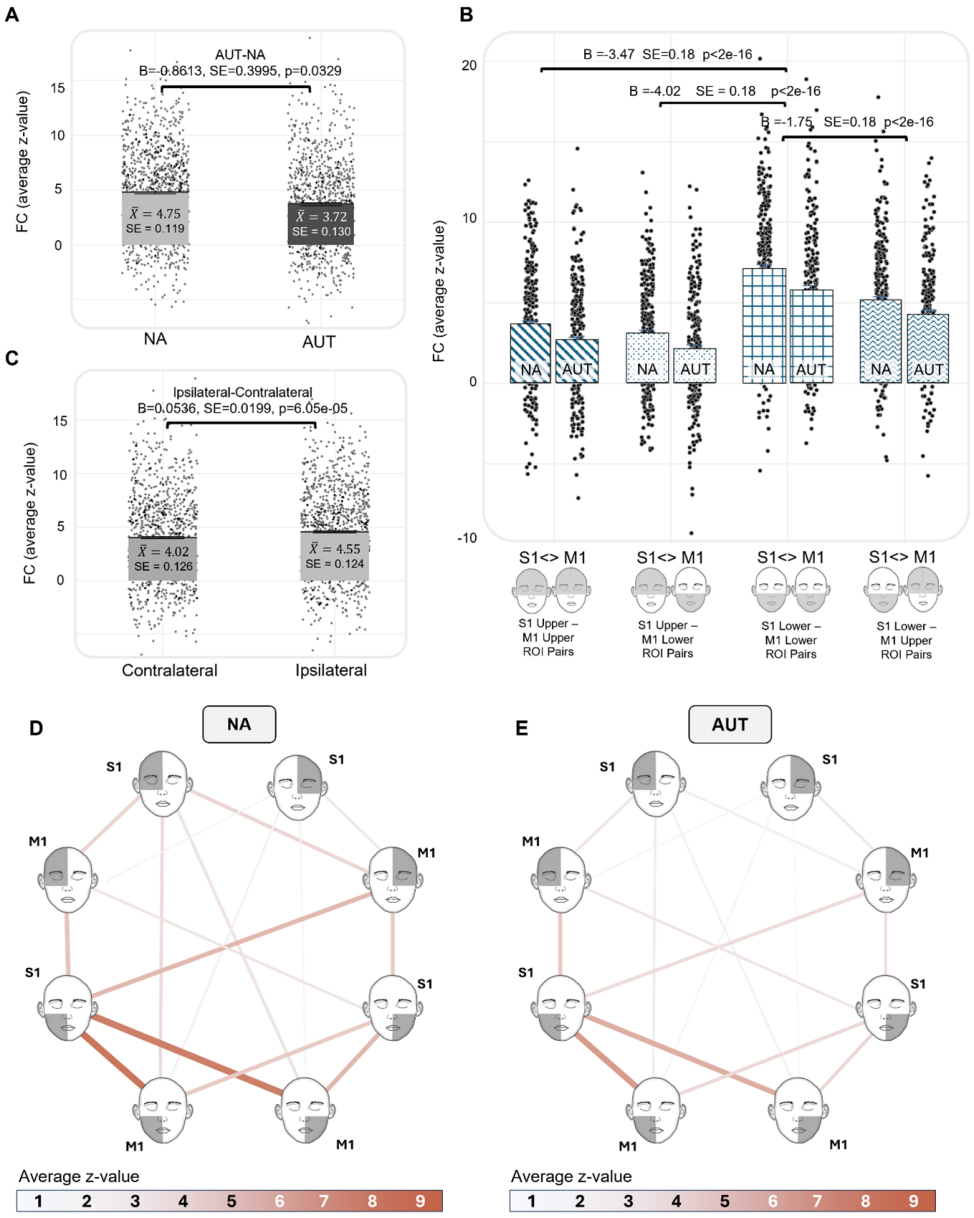
Vanderbilt Sample—Differences in functional connectivity across the divisions of upper–lower face half and laterality. (A) The main effect of group on FC (z-value). (B) The effect of upper–lower face half on FC, with S1 lower face—M1 lower face ROI pairs having significantly stronger FC than all other divisions. (C) The main effect of laterality (e.g., ipsilateral vs. contralateral ROI pairs) on FC. (D and E) (NA) and (AUT) illustrate the average z-value for each S1-M1 ROI pair, where thicker lines and more intense colors indicate higher values. These plots depict variations in connectivity across the upper–lower and left-right halves of the face. Additionally, they demonstrate that while we observed reduced overall connectivity in autism, the pattern of connectivity is similar across groups. There were no significant interactions between group, upper–lower face half, and laterality.

As observed in the previous model, in the Vanderbilt sample, FC was reduced in the AUT group compared with the NA group when considering both upper versus lower face half and laterality as fixed effects (FC ~ group + face half + laterality + age + sex + (1 | participant); [AUT-NA] B = -0.86137, SE = 0.39952, p = 0.03298; Fig. 6A). There were notable differences in FC across the upper and lower face, with S1 Lower Face-M1 Lower Face ROI pairs having significantly greater FC than S1 Upper Face-M1 Upper Face ROI pairs (B = -3.47, SE = 0.18, p < 2e-16), S1 Upper Face-M1 Lower Face (B = -4.02, SE = 0.18, p < 2e-16), and S1 Lower Face-M1 Upper Face (B = -1.75619, SE = 0.18, p < 2e-16) (Fig. 6B). In addition, ipsilateral ROI pairs were associated with greater FC than contralateral ROI pairs ([Ipsilateral–Contralateral] B = 0.53658, SE = 0.13349, p = 6.05e-05; Fig. 6C). Finally, increasing age was associated with greater FC (B = 0.05393, SE = 0.01993, p = 0.00775), while there was no significant difference in FC across genders ([F-M] B = 0.08275, SE = 0.44112, p = 0.85150).

To assess whether FC across the upper–lower face halves differed across groups, we re-ran the prior model first with an interaction term between diagnostic group and face half (FC ~ group*face half + laterality + age + sex + (1 | participant)) and then independently with an interaction term between diagnostic group and laterality (FC ~ group*laterality + face half + age + sex + (1 | participant)). There were no significant interactions between group and face half (Supplementary Table S5), nor between group and laterality (B = 0.12465, SE = 0.26809, p = 0.64203). This suggests that differences in S1-M1 FC across the upper–lower face follow similar patterns in the AUT and NA samples and that differences in ipsilateral and contralateral FC are consistent across groups. Finally, inclusion of a three-way interaction term between diagnostic group, upper–lower face half, and laterality yielded no significant effect (Supplementary Table S6). Model fit was compared using AIC and BIC values; the model including group, face half, and laterality as fixed effects without interaction terms had the lowest AIC (10839) and BIC (10895) values (Supplementary Table S2). Finally, for this best-fitting model, we performed Type II Wald chi-square tests to assess the contribution of each fixed effect. Face half (χ² (3) = 33.55, p < 0.0001), laterality (χ² (1) = 251.85, p < 0.0001), and age (χ² (1) = 5.23, p = 0.021) contributed significantly to the model, while diagnostic group (χ² (1) = 4.72, p = 0.2963) and gender (χ² (1) = 0.03, p = 0.8425) did not.

**Fig. 7.**
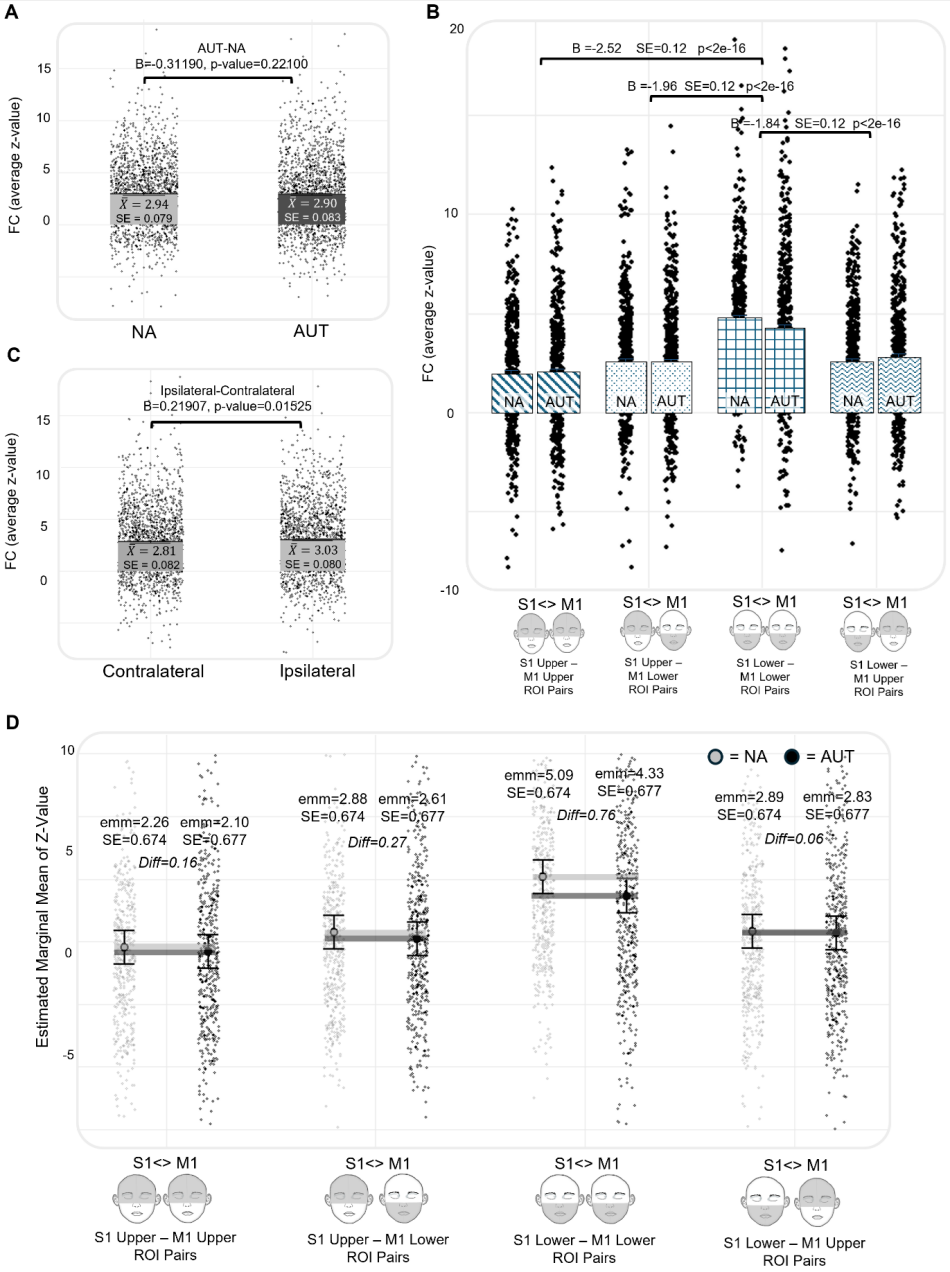
ABIDE Replication—Differences in functional connectivity across the divisions of upper–lower face half and laterality. Figure represents a partial replication of the results shown in Figure 6. (A) The main effect of group on FC (z-value). (B) The effect of upper–lower face half on FC, with S1 lower face—M1 lower face ROI pairs having significantly stronger FC than all other divisions, as was observed in the Vanderbilt sample. (C) The main effect of laterality (e.g., ipsilateral vs. contralateral ROI pairs) on FC. (D) The interaction effect of group and upper–lower face half on the EMM of z-value. The difference in EMM between groups [NA-AUT, SE NA = 0.674, SE AUT = 0.677] was significantly larger for S1 lower face–M1 lower face ROI pairs (5.09-4.33 = 0.76) than S1 upper face–M1 upper face (2.26-2.10 = 0.16), S1 upper face–M1 lower face (2.88-2.61 = 0.27), and S1 lower face–M1 upper face pairs (2.89-2.83 = 0.06).

When this analysis was repeated in the ABIDE sample, FC was not significantly different across groups ([AUT-NA] B = -0.31190, SE = 0.25409, p = 0.22100; Fig. 7A), representing a failure to replicate the main effect of group observed in the Vanderbilt sample. However, the pattern of FC among upper–lower face ROIs that was observed in the Vanderbilt sample was fully replicated in the ABIDE sample (Fig. 7B). In addition, there was a significant difference in FC across ipsilateral and contralateral ROI pairs ([Ipsilateral–Contralateral] B = 0.21907, SE = 0.09024, p = 0.01525; Fig. 7C). In the ABIDE sample, re-running the model to include an interaction term between diagnostic group and upper–lower face half revealed significant interactions between group and face half (Supplementary Table S5). We then derived EMMs from this model to compare the model-adjusted z-values across diagnostic group and across the upper–lower face halves; these differences in the EMM of z-value are shown in Figure 7D. Including an interaction term between diagnostic group and laterality revealed no significant interaction (B = 0.13377, SE = 0.18051, p = 0.45871), consistent with our findings in the Vanderbilt sample. Finally, including a three-way interaction term between group, face half, and laterality demonstrated no significant effect (Supplementary Table S6), consistent with our findings in the Vanderbilt sample. Model fit was compared using AIC and BIC values; the model including group, face half, and laterality as fixed effects without interaction terms had the lowest AIC (16938) and BIC (17012) values (Supplementary Table S3). Finally, for this best-fitting model, we again performed Type II Wald chi-square tests to assess the contribution of each fixed effect. Face half (χ² (3) = 218.83, p < 0.000001), laterality (χ² (1) = 8.45, p < 0.00365), and age (χ² (1) = 11.49, p < 0.00070) contributed significantly to the model, while diagnostic group (χ² (1) = 0.001, p = 0.9773) and gender (χ² (1) = 0.02, p = 0.8962) did not.

### 4.3 The relationship between facial expressivity and sensorimotor connectivity differs in autism

**Fig. 8.**
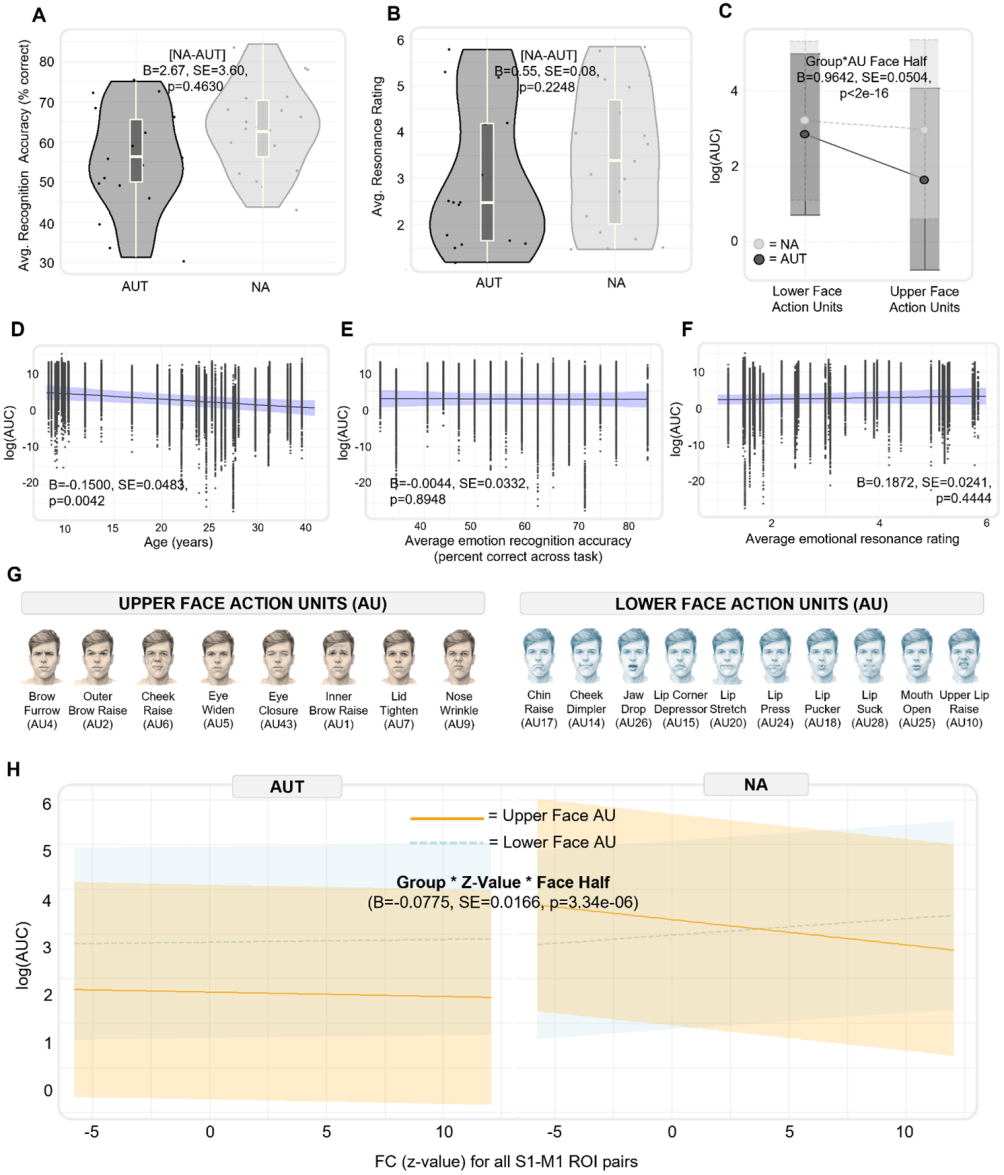
Association between group differences in facial expression behavior and S1-M1 functional connectivity (FC). (A and B) Group differences in the distribution of emotion recognition accuracy scores (percentage of correctly identified emotions across the 32 MET images) and mean emotion resonance ratings (self-reported Likert scores indicating how much the participant shared the depicted emotion). For emotion resonance, the plot shows the distribution of participant means for visualization only; statistical tests were performed using a linear mixed-effects model fit to all individual stimulus-level ratings (Eq. 3b). (C) Differences in the log-transformed area under the curve (log(AUC)) values, representing the integration of AU likelihood score over time, for lower face AUs versus upper face AUs across groups. We observed significant interaction between group and the upper–lower face halves, such that upper face AUs (but not lower face AUs) were more diminished in the AUT group than the NA group. (D) The main effect of age on log(AUC) values across all AUs, with older age being associated with decreased AU likelihood scores. (E and F) The effect of emotion recognition accuracy and emotional resonance ratings on log(AUC) values; neither had a significant effect. (G) The 18 individual action units (AU) classified by the iMotions AffDex engine and the face half (e.g., upper or lower) they were assigned to in this analysis (images by Jure Blom, used with permission—https://www.jurreblom.nl/). (H) The significant three-way interaction effect of group, FC (z-value), and AU face half on log(AUC) value, demonstrating that the association between FC and AU likelihood varies across groups as well as across the upper versus lower face halves.

Among participants who completed the MET-J, there were no significant group differences in emotion recognition accuracy (Eq. 3a; B = 2.67, SE = 3.60, p = 0.4630; Fig. 8A) or in self-reported emotional resonance ratings (Eq. 3b; B = 0.55, SE = 0.08, p = 0.2248; Fig. 8B). Despite this, we included emotion recognition accuracy and average emotional resonance as covariates in all subsequent models to test the extent to which these variables might account for group differences in facial expression behavior. For each of the 32 MET-J stimuli, we calculated the log(AUC) values for 8 upper face AUs and 10 lower face AUs. We modeled the main effects of group and AU face half (upper vs. lower) on log(AUC) values, including age, gender, emotion recognition accuracy, and emotional resonance as covariates. We also accounted for repeated measures by modeling participant, stimulus, and AU as random intercepts (Eq. 4). There was no main effect of group (B = 0.3611, SE = 0.6829, p = 0.6010) nor AU face half (B = -1.185, SE = 1.242, p = 0.3526) on log(AUC) values. However, when we refit the model including an interaction term between group and AU face half, this interaction was significant (B = 0.9642, SE = 0.0504, p < 2e-16; Fig. 8C). In the NA group, log(AUC) values were similar for the upper and lower face AUs. However, in the AUT group, log(AUC) values for upper face AUs were significantly less than lower face AUs, suggesting reduced expressivity specific to the upper face.

In this interaction model, age was associated with decreased log(AUC) values (B = -0.1500, SE = 0.0483, p = 0.0042; Fig. 8D), while gender had no significant effect (B = 0.6214, SE = 0.8361, p = 0.4633). Finally, neither emotion recognition accuracy (B = -0.0044, SE = 0.0332, p = 0.8948; Fig. 8E) nor self-reported emotional resonance (B = 0.1872, SE = 0.2414, p = 0.4444; Fig. 10F) significantly predicted log (AUC) values, indicating that group differences in facial expression behavior across the upper and lower face were not explained by individual variation in emotional recognition or emotional resonance.

Finally, to assess whether log(AUC) values were associated with FC among the S1-M1 face ROIs and whether this effect differed by group and AU face half, we refit the prior model, adding FC (z-value among all 16 S1-M1 ROI pairs) as a fixed effect as well as a three-way interaction between diagnostic group, AU face half, and FC. To account for repeated measures introduced by the 16 unique ROI pairs per participant, we included an additional random intercept for ROI pair (log(AUC) ~ group* AU face half*FC + age + gender + (1| participant) + (1| stimulus) + (1| AU) + (1| ROI pair).

There was no main effect of diagnostic group (B = 0.1621, SE = 0.6867, p = 0.8149), FC (B = 0.0061, SE = 0.0081, p = 0.4481), nor AU face half (B = -1.1170, SE = 1.241, p = 0.3799) on log(AUC) values. However, as in the prior model, there was a significant interaction between diagnostic group and AU face half (B = 1.4670, SE = 0.1014, p < 2e-16). In the NA group, there was no significant difference in log(AUC) values between upper and lower face AUs, while in the AUT group, log(AUC) values for upper face AUs were significantly less than those for the lower face. There was a significant interaction between diagnostic group and FC (B = 0.0307, SE = 0.0119, p = 0.0099), but not between AU face half and FC (B = -0.0160, SE = 0.0116, p = 0.1684). Finally, we observed a significant three-way interaction between group, FC, and AU face half (B = -0.0775, SE = 0.0166, p = 3.34e-06; Fig. 8H). This interaction suggests that the relationship between FC and AU likelihood differs across groups and across the upper and lower face. Specifically, in the NA group, there was a negative association between FC and log (AUC) values for upper face AUs, while a positive association was found for lower face AUs. This indicates that S1-M1 FC is differentially related to facial expression behavior across the upper and lower face. In contrast, the AUT group did not show this moderating effect of upper–lower face half, suggesting a more uniform relationship between FC (z-value) and facial expression behavior (AU likelihood) across the upper and lower face.

## 5 Discussion

Facial expressions are unique sensorimotor behaviors in which fine muscle contractions across the upper and lower face occur in a coordinated, precise fashion to manipulate the superficial facial features (e.g., eyebrows, nose, cheeks, lips) into prototypical configurations (Bress & Cascio, 2024). Accurate execution of this sensorimotor sequence is necessary to reliably produce facial expressions that appear contextually appropriate in magnitude, duration, and timing. Here, we present novel, empirical evidence that (1) FC between the primary somatosensory and primary motor cortices has meaningful, replicable somatotopic topology which reflects the distinct neuroanatomic pathways underlying motor control of the upper and lower face, (2) the pattern of this topology is largely preserved in autism, although its strength may be weakened, (3) autism is associated with diminished expression, specifically in the upper face, and (4) this diminished upper face expressivity in autism is associated with reduced S1-M1 connectivity relative to a nonautistic comparison group.

In this work, we utilized rs-fMRI data from two independent samples to characterize the topology of functional connections between the somatotopic face areas of S1 and M1. We demonstrate *and replicate* that while functional connectivity between these cortical areas follows a somatotopic pattern, this pattern is specific and limited to the lower face. In both the Vanderbilt (n = 71 NA) and ABIDE (n = 109 NA) samples, upper face areas of S1 had equivalent FC with both the upper and lower face areas of M1, while the lower face areas of S1 had greater FC with the lower face areas of M1 than the upper face areas of M1. These patterns suggest that upper face sensory information influences both upper and lower face motor control in a nonspecific manner, whereas lower face cortical sensorimotor connectivity is somatotopically specific. This may reflect a central mechanism supporting more precise sensorimotor feedback for lower face motor functions. Given the lower face’s involvement in many other complex sensorimotor tasks such as mastication and speech, the lower face likely requires more specific and robust sensorimotor feedback than the upper face. Additionally, this finding of somatotopic organization specific to the lower face may reflect well-known differences in the lateral redundancy of upper versus lower face motor control (Benecke et al., 1988; Ross et al., 2016). While upper face muscles receive input from both ipsilateral and contralateral cortical projections to the medial facial motor brainstem subnucleus, lower face muscles rely heavily on contralateral cortical inputs to the lateral facial motor subnucleus (Jenny & Saper, 1987; Pilurzi et al., 2013). Thus, our findings provide functional evidence that facial expression, even at the level of the S1 and M1, requires a specific, organized feedback loop through which sensory information influences motor control. Disruptions in this specificity, particularly for motor systems without redundancy, may result in disorganized execution of sensorimotor behaviors.

Beyond characterizing the typical topology of FC across the facial somatosensory and motor cortices, the goal of this work was to investigate whether and how this connectivity may differ in autism. Using multiple linear mixed effects models, we assessed the relationship between FC in a matrix of ROI pairs composed of the somatotopic face regions of S1 and M1. In both the Vanderbilt (n = 130, n = 59 AUT) and ABIDE (n = 218, n = 109 AUT) samples, we observed the same topology of FC across the autistic and nonautistic samples. This includes somatotopic specificity for the lower face, as well as a lack of such specificity for the upper face. However, there was some evidence for diminished overall connectivity in autism (Vanderbilt sample), and for less connectivity in autism specific to the S1-M1 lower face representations (ABIDE sample). Taken together, these findings suggest that while the patterns we observe are consistent across groups in their quality, they may be somewhat attenuated in autism.

Our goal in investigating a sensorimotor basis for facial expressivity differences in autism is to interpret potential differences in FC within the context of behavior. Here, we utilize a behavioral paradigm involving passive viewing of emotionally charged images to elicit spontaneous facial expression in both autistic (n = 17) and nonautistic (n = 19) individuals, with facial expression behavior operationalized as automatically detected and coded facial action units (AUs). We report that, in response to 32 distinct emotional stimuli, AUT individuals showed reduced facial expression in the upper face compared with the lower face—a pattern that was not observed in the NA group. This is particularly relevant given that autistic facial expressions are often rated as unusual in quality by nonautistic individuals (Trevisan et al., 2018), and the upper face plays a key role in relatively subtle components of genuine facial expressions such as the tightening of eyelids and raising of cheeks in a “Duchenne” smile (Soussignan, 2002; Webster et al., 2021). These upper face actions likely have a strong impact on the subjective quality of facial expressions. Furthermore, facial expression components which are commonly disrupted in autism, including difficulty pairing facial expressions with eye contact to appropriately direct them toward social partners, localize to the upper face.

To examine the neurobiological and behavioral components of our study together, we tested the effects of FC, diagnostic group, and upper–lower face half on facial expression behavior. In the NA group, S1-M1 FC was differentially associated with expressivity across the upper and lower face: greater S1-M1 FC was linked to increased activation of lower face AUs and a decreased activation of upper face AUs. This pattern was not present in the AUT group, where FC showed a uniformly flat association with AU scores for both the upper and lower face. These findings suggest that, in NA individuals, S1-M1 FC may play distinct roles in modulating expressivity in the upper versus lower face. This is consistent with well-documented neuroanatomical features unique to upper face motor control, namely the bilaterality of corticobulbar inputs to the upper-face musculature (Bress & Cascio, 2024; Hopf et al., 1992; Rinn, 1984) and the more prominent contribution of subcortical pathways, such as the amygdala and basal ganglia, in supporting spontaneous activation of upper-face muscles (Gothard, 2014; Weddell, 1994). Considered jointly, the markedly diminished use of the upper face and the absence of a differential association between S1-M1 FC and facial expressivity in the AUT group may reflect disrupted coordination between cortical and subcortical drivers of upper face motor control. As many—if not all—naturalistic facial expressions require coordinated activity across the upper and lower halves of the face, this disruption may contribute to the decreased cohesiveness of facial expressions that is commonly described in autism (Trevisan et al., 2018).

Finally, by including measures of emotion recognition accuracy and self-reported emotional resonance in our analyses, we demonstrate that group differences in facial expression behavior—and their association with FC—persist even when accounting for individual variability in these factors. Though these findings absolutely require replication in larger samples, they challenge the long-held assumption that facial expressivity differences in autism are solely driven by disruptions in social cognition (e.g., emotion recognition, salience, or empathy) and instead point toward a potential contribution of sensorimotor mechanisms. Notably, atypical facial expressivity may itself shape social cognition differences in autism, as disruptions in emotional embodiment processes such as facial mimicry are shown to diminish the generation, perception, and sharing of emotional states (Chartrand & Lakin, 2013; Hirsch et al., 2023). Thus, clarifying the sensorimotor basis of atypical expressivity in autism is critical for understanding facial expression not only as a social communication behavior, but also as a mechanism of emotion simulation, empathy, and embodied cognition—processes fundamental to reciprocal social engagement.

## 6 Conclusions and Limitations

In conclusion, this work presents clear evidence that further investigation of a sensorimotor basis for facial expressivity differences in autism is warranted and suggests that differences in the balance of FC across the upper–lower face axis may be a driver of facial expressivity differences in autism. To this end, we advocate that future investigations in this arena must consider the upper and lower face distinctly. Composite or summary metrics of facial expressivity, such as those utilized in prior work from our laboratory (J. Quinde-Zlibut et al., 2022), might obscure these subtle but important distinctions and thus mask nuanced group differences that are critical for understanding the mechanisms driving atypical facial expression behavior. There are several other key limitations of this work that future research can address, including the lack of participant-specific, functional definition of the facial sensory and motor regions. Precise mapping of the facial sensorimotor brain network will require the application of task-based functional paradigms for this purpose. Moreover, as resting-state FC is inherently correlational, this work cannot elucidate the important and still unanswered question of how facial sensory information reaches or is integrated into motor control systems. Our findings reflect nondirectional connectivity among the facial sensory and motor systems that likely involves both top–down and experience-dependent feedback mechanisms. Future application of causal inference frameworks to delineate effective connectivity is necessary for truly characterizing the mechanisms of sensorimotor control for facial expression.

Finally, it must be noted that while the MET-J is validated for assessing cognitive and emotional empathy, it is not specifically validated as a measure of facial expressivity. However, the MET-J employs a standardized stimulus set (e.g., IAPS) and a validated facial coding system (e.g., FACS), both of which are well-established tools for eliciting and quantifying facial expressions. Because there remains no standardized task for measuring facial expressivity, we leverage the MET-J to capture both participants’ empathic responses to emotional images and their spontaneous facial reactions to those images, capitalizing on its use of standardized stimuli and validated facial action coding methods. Although a variety of stimuli can elicit spontaneous facial expressivity, evidence suggests that static images—such as those in the MET-J—are less effective than dynamic stimuli for eliciting naturalistic, spontaneous facial expressions (Krumhuber et al., 2013). Furthermore, naturalistic facial expressions often reflect a blend of spontaneous and posed elements, a distinction that the current paradigm does not address. Careful experimental design will be necessary to operationalize the measurement of facial expressivity across these nuanced and complex domains. Additionally, while demographic factors such as sex and age are known to influence facial expressivity, our study was underpowered—particularly in the behavioral sample—to adequately examine these effects. Future work in both clinical and non-clinical samples should be designed with sufficient power to consider the impact of demographic variables on facial expressivity.

It is certain that facial expression involves many brain regions beyond S1 and M1 and has known subcortical drivers not explored in this work, especially cingulomotor and subcortical drivers that integrate sensorimotor control with limbic regions representing affective significance (Gothard, 2014). Future work must assess how accessory sensory and motor areas, as well as subcortical structures (Gothard, 2014), contribute to the sensorimotor circuits regulating facial motor control from the cortex to the periphery. This work is an initial step toward a mechanistic understanding of facial expressivity differences in autism to inform evidence-based design of therapeutic approaches, as well as challenging the misconception that atypical facial expressivity in autism is solely or even primarily attributable to differences in social motivation (Jaswal & Akhtar, 2019; Kapp et al., 2019). Social communication behaviors such as facial expression involve complex motor coordination depending on unique and precise sensory feedback; we must not neglect how the sensory and motor domains of autism phenomenology compound and potentially drive differences in social behavior which have been long attributed to differences in social or emotional competence alone.

## Supplementary Material

Supplementary Material

## Data Availability

Anonymized data are available upon request. Data from the Autism Brain Imaging Data Exchange can be retrieved from the NeuroImaging Tools and Resource Collaboratory (https://www.nitrc.org/). Region of Interest (ROI) mask files can be accessed at (https://github.com/bressks1/s1_m1_face_roi_masks). Our code for preprocessing of resting-state fMRI data and production of functional connectivity matrices can be accessed at https://github.com/baxpr/connprep and https://github.com/baxpr/conncalc, respectively.
