# Supplementary material for: A sensorimotor basis for facial expressivity differences in autism
Source: Imaging Neurosci (Camb). 2025 Nov 10;3:IMAG.a.981. doi: 10.1162/IMAG.a.981 (PMC12603653; doi:10.1162/IMAG.a.981)
Supplement: Supplementary Material [file IMAG.a.981_supp.pdf]

Table 1: MNI Coordinates for somatotopic body areas of the primary somatosensory (S1) and motor (M1) cortices, as reported by Roux et al. We built 4mm spheres around these MNI coordinates, originally reported by Roux et al. 2018 (S1) and 2020 (M1). Spheres were then aggregated to form the composite S1 and M1 face areas utilized as regions of interest (ROI) in this analysis. ROI masks can be accessed at: https://github.com/bressks1/s1_m1_face_roi_masks.

Composite Region of Interest	Body Areas Represented	Central MNI Coordinate for each body area
**Composite S1 Regions of Interest**		
	Forehead	-43.5 -9.0 54.0
	Eyes	-52.0 -6.0 45.0
Left Upper Face	Eyebrows	-45.0 -6.0 53.0
	Nose	-44.0 -7.0 54.0
	Cheek	-50.4 -2.8 44.2
	Forehead	43.5 -9.0 54.0
	Eyes	52.0 -6.0 45.0
Right Upper Face	Eyebrows	45.0 -6.0 53.0
	Nose	44.0 -7.0 54.0
	Cheek	50.4 -2.8 44.2
Right Lower Face	Lips	55.0 -1.4 37.4
	Jaw	59.8 -0.3 23.2
Left Lower Face	Lips	-55.0 -1.4 37.4
	Jaw	-59.8 -0.3 23.2
**Composite M1 Regions of Interest**		
	Eyebrows	-56.0 -21.0 46.0
Left Upper Face	Eyes	-53.0 -23.0 48.0
	Nose	-56.0 -19.0 45.0
	Cheek	-55.0 -15.0 44.0
	Eyes	56.0 -21.0 46.0
Right Upper Face	Eyebrows	53.0 -23.0 48.0
	Nose	56.0 -19.0 45.0
	Cheek	55.0 -15.0 44.0
Right Lower Face	Lips	57.5 -13.3 41.9
	Jaw	60.5 -13.0 37.5
Left Lower Face	Lips	-57.5 -13.3 41.9
	Jaw	-60.5 -13.0 37.5

Table 2: Comparison of model fit and variance – Vanderbilt sample

Model[a]	AIC	BIC	Total variance	Residual variance (SD)	Variance explained by Participant (SD)
z-value~ group + somatotopic identity	11076	11133	14.83397	10.486 (3.238)	4.348 (2.085)
z-value~ group*somatotopic identity	11080	11159	14.83397	10.500 (3.240)	4.347 (2.085)
z-value~ group + face half + laterality	10839	10895	13.68461	9.260 (3.043)	4.424 (2.103)
z-value~ group*face half + laterality	10844	10917	13.68961	9.266 (3.044)	4.424 (2.103)
z-value~ group*laterality + face half	10841	10903	13.68811	9.264 (3.044)	4.424 (2.103)
z-value~ group*face half*laterality	10850	10962	13.68902	9.265 (3.044)	4.424 (2.103)

[a]All models also include gender and age as fixed effect, as well as participant identity as a random effect

Table 3: Comparison of model fit and variance – ABIDE sample

Model[a]	AIC	BIC	Variance	Residual Variance (SD)	***Variance explained by:***		
					**Participant (SD)**	**Site (SD)**	**Eye status (SD)**
z-value~ group + somatotopic identity	17148	17222	11.4235	7.4855 (2.7360)	2.8422 (1.6859)	0.5690 (0.7543)	0.5269 (0.7259)
z-value~ group*somatotopic identity	17151	17249	11.4209	7.4823 (2.7354)	2.8424 (1.6959)	0.5690 (0.7544)	0.5269 (0.7259)
z-value~ group + face half + laterality	16938	17012	10.9715	7.0034 (2.6262)	2.8723 (1.6948)	0.5690 (0.7543)	0.5268 (0.7258)
z-value~ group*face half +laterality	16941	17021	10.9590	7.0034 (2.6262)	2.8732 (1.6950)	0.5689 (0.7543)	0.5270 (0.7259)
z-value~ group*laterality + face half	16938	17030	10.9715	7.0034 (2.6262)	2.8722 (1.6948)	0.5689 (0.7542)	0.5273 (0.7262)
z-value~ group*face half*laterality	16954	17089	10.9681	7.0034 (2.6262)	2.8727 (1.6949)	0.5689 (0.7542)	0.5272 (0.7261)

[a]All models also include gender and age as fixed effect, as well as participant identity, site, and eye status as random effects

Table 4: Somatotopic identity contrasts conditioned on group - Vanderbilt and ABIDE Samples

ROI Pair Contrasts	Group	Estimate	SE	df	t ratio	p-value
**Vanderbilt Sample**						
Left S1 Upper Face <> Left M1 Upper Face - Non-somatotopic	AUT-NA	-0.0585	0.5941	1942	-0.0990	0.9214
Right S1 Upper Face <> Right M1 Upper Face - Non-somatotopic	AUT-NA	0.4649	0.5941	1942	0.7830	0.4340
Left S1 Lower Face <> Left M1 Lower Face – Non-somatotopic	AUT-NA	-0.2722	0.5941	1942	-0.4580	0.6468
Right S1 Lower Face <> Right M1 Lower Face - Non-somatotopic	AUT-NA	-0.4624	0.5941	1942	-0.7780	0.4364
**ABIDE Sample**						
Left S1 Upper Face <> Left M1 Upper Face - Non-somatotopic	AUT-NA	0.2547	0.3883	3217	0.6560	0.5119
Right S1 Upper Face <> Right M1 Upper Face - Non-somatotopic	AUT-NA	0.3501	0.3883	3217	0.9020	0.3673
Left S1 Lower Face <> Left M1 Lower Face – Non-somatotopic	AUT-NA	-0.2409	0.3883	3217	-0.6200	0.5350
Right S1 Lower Face <> Right M1 Lower Face - Non-somatotopic	AUT-NA	-0.6982	0.3883	3217	-1.7980	0.0722

Table 5: Face half contrasts conditioned on group - Vanderbilt and ABIDE Samples

ROI Pair Contrasts	Group	Estimate	SE	df	t ratio	p-val
**Vanderbilt Sample**						
S1 Lower <> M1 Upper - S1 Lower <> M1 Lower	AUT-NA	0.4669	0.3791	1943	1.2310	0.2183
S1 Upper <> M1 Lower - S1 Lower <> M1 Lower	AUT-NA	0.3900	0.3791	1943	1.0290	0.3037
S1 Upper <> M1 Upper - S1 Lower <> M1 Lower	AUT-NA	0.3999	0.3791	1943	1.0550	0.2917
**ABIDE Sample**						
S1 Lower <> M1 Upper - S1 Lower <> M1 Lower	AUT-NA	0.7092	0.2550	3218	2.781	0.0054
S1 Upper <> M1 Lower - S1 Lower <> M1 Lower	AUT-NA	0.5014	0.2550	3218	1.966	0.0435
S1 Upper <> M1 Upper - S1 Lower <> M1 Lower	AUT-NA	0.6125	0.2550	3218	2.402	0.0163

Table 6: Face half contrasts conditioned on group and laterality – Vanderbilt and ABIDE samples

ROI Pair Contrasts	Group	Laterality	Estimate	SE	df	t ratio	p-value
**Vanderbilt Sample**							
S1 Lower <> M1 Upper – S1 Lower <> M1 Lower	AUT-NA	Ipsilateral-Contralateral	-0.2650	0.7583	1936	0.350	0.7267
S1 Upper <> M1 Lower – S1 Lower <> M1 Lower	AUT-NA	Ipsilateral-Contralateral	-0.1531	0.7583	1936	0.202	0.8399
S1 Upper <> M1 Upper – S1 Lower <> M1 Lower	AUT-NA	Ipsilateral-Contralateral	-0.3412	0.7583	1936	0.450	0.6527
**ABIDE Sample**							
S1 Lower <> M1 Upper – S1 Lower <> M1 Lower	AUT-NA	Ipsilateral-Contralateral	-0.0421	0.5103	3211	-0.083	0.9342
S1 Upper <> M1 Lower – S1 Lower <> M1 Lower	AUT-NA	Ipsilateral-Contralateral	-0.1171	0.5103	3211	-0.230	0.8184
S1 Upper <> M1 Upper – S1 Lower <> M1 Lower	AUT-NA	Ipsilateral-Contralateral	-0.3189	0.5103	3211	-0.625	0.5320
